# Immunogenicity of AS03-adjuvanted and non-adjuvanted trivalent inactivated influenza vaccines in elderly adults: A Phase 3, randomized trial and *post-hoc* correlate of protection analysis

**DOI:** 10.1080/21645515.2016.1219809

**Published:** 2016-10-03

**Authors:** Guillermo M. Ruiz-Palacios, Geert Leroux-Roels, Jiri Beran, Jeanne-Marie Devaster, Meral Esen, Odile Launay, Janet E. McElhaney, Gerrit A. van Essen, Anne Benoit, Carine Claeys, Walthère Dewé, Christelle Durand, Xavier Duval, Ann R. Falsey, Gregory Feldman, Florence Galtier, Pierre Gervais, Shinn-Jang Hwang, Shelly McNeil, Jan Hendrik Richardus, Andrew Trofa, Lidia Oostvogels, for the Influence65 study group

**Affiliations:** a Department of Infectious Diseases, Instituto Nacional de Ciencias Médicas y Nutrición Salvador Zubirán, Tlalpan, C.P. México City, México; b Centre for Vaccinology, Ghent University and Ghent University Hospital, Ghent, Belgium; c Vaccination and Travel Medicine Centre, Hradec Kralove, Czech Republic; and 2nd Faculty of Medicine, Charles University in Prague, Czech Republic; d Jeanne-Marie Devaster, MD, GSK Vaccines, Rixensart, Belgium; e Institut für Tropenmedizin, Tübingen, Germany; f Inserm, CIC 1417 and French Network of Clinical Investigation in Vaccinology (I-REIVAC), France; Université Paris Descartes, Sorbonne Paris Cité, Paris, France; Assistance Publique-Hôpitaux de Paris (AP-HP), Hôpital Cochin, CIC Cochin Pasteur, Paris, France; g HSN Volunteer Association Chair in Geriatric Research, Health Sciences North Research Institute, Sudbury, ON, Canada; h Julius Center for Health Sciences and Primary Care, University Medical Center Utrecht, Utrecht, The Netherlands; i Institute of Statistics, Biostatistics and Actuarial Sciences, Université Catholique de Louvain, Louvain-la-Neuve, Belgium; j GSK Vaccines, Wavre, Belgium; k GSK Vaccines, Rixensart, Belgium; l GSK Vaccines, Wavre, Belgium; m Hôpital Bichat Claude Bernard, GH BICHAT. Paris cedex 18, France; Inserm, CIC 007 for the French Network of Clinical Investigation in Vaccinology (REIVAC), Paris Cedex 18, France; n University of Rochester Medical Center, Rochester General Hospital, Rochester, NY, USA; o Carolina Pharmaceutical Research, Spartanburg, South Carolina, United States; p CHRU de Montpellier, Hôpital Saint Eloi, Montpellier, France; Inserm, CIC 1001 for the French Network of Clinical Investigation in Vaccinology (REIVAC), Montpellier, France; q Q&T Research Sherbrooke, Sherbrooke, QC, Canada; r Department of Family Medicine, Taipei Veterans General Hospital, Taipei, Taiwan; National Yang-Ming University School of Medicine, Taipei, Taiwan; s Queen Elizabeth Health Sciences Centre, Dalhousie University, PCIRN, NACI, CCfV, CAIRE, QEII HSC - VG Site Infectious Diseases, Halifax, Nova Scotia, Canada; t GGD Rotterdam-Rijnmond, Rotterdam, The Netherlands; u GSK Vaccines, King of Prussia, PA, USA; v GSK Vaccines, Wavre, Belgium

**Keywords:** AS03, correlates of protection, immunogenicity, older, seasonal influenza, vaccine

## Abstract

In this study we describe the immunogenicity results from a subset of older people (N = 5187) who participated in a Phase 3 randomized, observer-blinded trial of AS03-TIV versus TIV (*Fluarix*™) (ClinicalTrials.gov, NCT00753272). Participants received one dose of AS03-TIV or TIV in each study year and antibody titers against the vaccine strains were assessed using hemagglutination-inhibition (HI) assay at 21 d and 180 d post-vaccination in each vaccine group in the 2008/09 (Year 1) and 2009/10 (Year 2) influenza seasons. Manufacturing consistency of 3 lots of AS03-TIV for HI antibody responses in Year 1 was a co-primary objective.

In a *post-hoc* analysis, a statistical regression model included 4830 subjects in whom immunogenicity and laboratory-confirmed attack rate data were available; the analysis was performed to assess HI antibody titers against A/H3N2 as a correlate of protection for laboratory-confirmed A/H3N2 influenza.

AS03-TIV and TIV elicited strong HI antibody responses against each vaccine strain 21 d post-vaccination in both years. The manufacturing consistency of 3 lots of AS03-TIV was demonstrated. In both years and each vaccine group, HI antibody responses were lower for A/H1N1 than the other vaccine strains. Day 180 seroconversion rates (proportion with ≥4-fold increase in titer compared with pre-vaccination titer) in Year 1 in the AS03-TIV and TIV groups, respectively, were 87.7% and 74.1% for A/H3N2, 69.7% and 59.6% for influenza B, and 58.3% and 47.4% for A/H1N1.

The *post-hoc* statistical model based on A/H3N2 attack rates and HI antibody titers estimated that a 4-fold increase in post-vaccination titers against A/H3N2 was associated with a 2-fold decrease in the odds of A/H3N2 infection.

## Introduction

Observational studies suggest that the effectiveness of trivalent inactivated influenza vaccine (TIV) is reduced in older people compared with younger populations, and this is thought to be associated with age-related decline in immune functions, which impairs the ability to resist influenza infection and respond to vaccination.^1,2^ However, because it is unethical to use a placebo vaccine in high-risk populations, reliable estimates of absolute efficacy of existing influenza vaccines in older people are lacking.^3^ Strategies to improve the immunogenicity of TIVs with the aim of reducing influenza-related morbidity and mortality in older people includes the use of high-doses of hemagglutinin antigen (HA), intradermal administration, and formulation with adjuvant systems.^4-8^

Although newer influenza vaccine formulations for use in older people have been shown to increase immunogenicity,^4-8^ the vaccine efficacy of candidate vaccines can only be assessed relative to the existing standard of care. Most recently, in a randomized study of 31,989 people aged ≥65 years, high-dose (180 µg HA) vs. standard dose (45 µg HA) TIV (*Fluzone™*; Sanofi Pasteur) was found to be better for the prevention of any influenza infection, with a relative efficacy of 24.2%.^9^ However, in a randomized, multinational trial of 43,000 people aged ≥65 years (*Influence65* trial), AS03-adjuvanted TIV (AS03-TIV) versus TIV (*Fluarix™*; GlaxoSmithKline) did not significantly prevent influenza A and/or B, with a relative efficacy of 12.0%.^10^ The predominant virus in the *Influence65* trial was A/H3N2, and *post-hoc* analyses showed significant relative efficacy for AS03-TIV vs. TIV for the prevention of influenza A/H3N2 infection-related clinical outcomes including all-cause death and pneumonia. The relative vaccine efficacy for AS03-TIV versus TIV for the prevention of A/H3N2 was 22.0% (95% CI: 5.68 to 35.49).^10^

Here we describe the immunogenicity for AS03-TIV vs. TIV in people aged ≥65 years based on a subset of the population from the Phase 3 *Influence65* trial. In addition, we assessed the relationship between A/H3N2 infection rates and vaccine-induced antibody titers against A/H3N2 to evaluate the HI antibody titers as a correlate of vaccine efficacy.

## Results

### Study population

**Figure 1.**
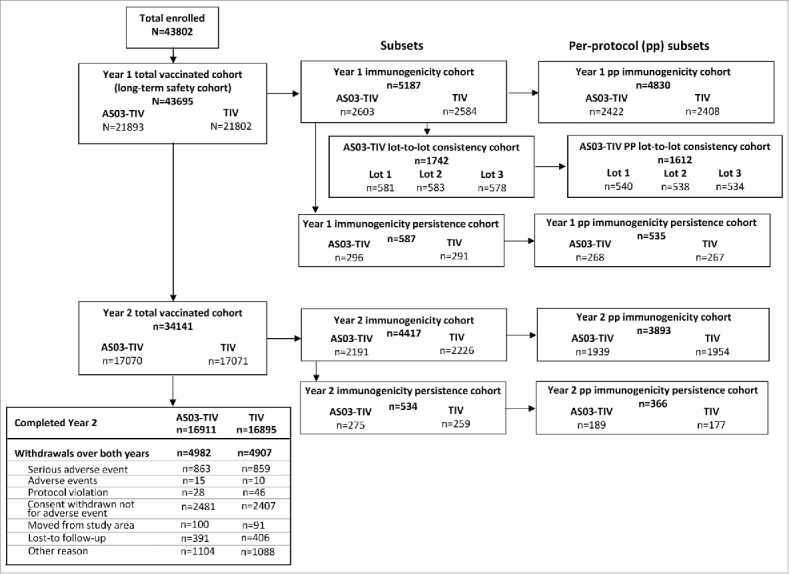
Participant flow chart. Note: AS03, tocopherol, oil-in-water emulsion-based Adjuvant System; CI, confidence intervals; TIV, inactivated trivalent influenza vaccine; Year 1, 2008/09; Year 2, 2009/10.

The immunogenicity subset included 5187 and 4417 subjects in Year 1 and 2, respectively (Fig. 1). In this subset, the mean age at first vaccination was 73.2 y (range 65–95 years) in the AS03-TIV group, and 73.4 y (range 65–100 years) in the TIV group.

In the total vaccinated cohort of the *Influence65* trial, a total of 43,695 subjects were vaccinated in Year 1 and 34,141 also received a second vaccination in Year 2. The study was initiated on 15 September 2008 and the data lock point in Year 1 was 23 April 2010, and in Year 2 the data lock point was 3 May 2011, respectively. Study cohorts and reasons for withdrawal from the total vaccinated cohort are shown in Figure 1.

### Lot-to-lot consistency

The AS03-TIV per-protocol consistency cohort comprised 1612 subjects of which 540 received lot 1, 538 lot 2, and 534 lot 3. At Day 21 after the first AS03-TIV vaccination, the 2-sided 95% Confidence Interval (CI) of the adjusted geometric mean titer (GMT) ratios for each lot-to-lot comparison was within the pre-defined interval for consistency for each vaccine strain (Table 1). The seroconversion rate (SCR) on Day 21 in subjects who received AS03-TIV lot 1, 2, and 3 ranged from 55.6% to 58.3% against A/H1N1, from 85.5% to 87.4% against A/H3N2 and from 65.3% to 72.2% against the B strain.

**Table 1.** Hemagglutination-inhibition-based adjusted GMT ratios at Day 21 after vaccination for 3 lots of AS03-TIV in the per-protocol consistency cohort.

	Adjusted GMT		Adjusted GMT ratio (95% CI)
	Lot 1 (N = 539)	Lot 2 (N = 536)	Lot 1/lot 2
A/H1N1	82.6	83.8	0.99 (0.87, 1.12)
A/H3N2	271.9	287.5	0.95 (0.82, 1.09)
B strain	649.0	600.8	1.08 (0.97, 1.20)
	Lot 2 (N = 536)	Lot 3 (N = 532)	Lot 2/lot 3
A/H1N1	83.7	93.6	0.89 (0.78, 1.02)
A/H3N2	283.7	271.8	1.04 (0.90, 1.21)
B strain	594.4	605.6	0.98 (0.88, 1.09)
	Lot 1 (N = 539)	Lot 3 (N = 532)	Lot 1/lot 3
A/H1N1	82.6	93.6	0.88 (0.78, 1.00)
A/H3N2	269.8	273.4	0.99 (0.85, 1.14)
B strain	646.2	609.9	1.06 (0.95, 1.18)

Adjusted GMT, geometric mean titer adjusted for baseline titer of the 2 compared lots; AS03, tocopherol-based oil-in-water emulsion Adjuvant System; CI, confidence interval; TIV, inactivated trivalent influenza vaccine; N, number of subjects with pre- and post-vaccination results available.

### Immunogenicity at Day 0 and Day 21

Before vaccination in Year 1, in the AS03-TIV and TIV groups, respectively, 69.3% and 68.0% were seropositive (titer ≥1:10) for A/H1N1, 64.7% and 65.0% were seropositive for A/H3N2, and 95.0% and 94.2% were seropositive for the influenza B strain included in the vaccine (Victoria lineage).

**Figure 2.**
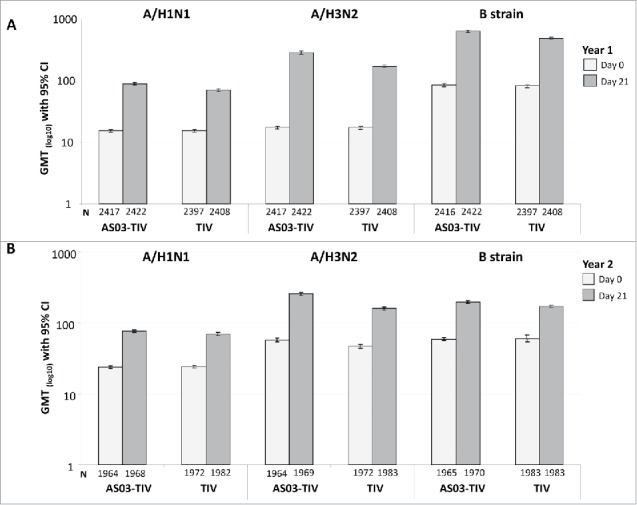
Day 21 hemagglutination-inhibition-based GMTs in the per-protocol immunogenicity cohort in Year 1 (A) and Year 2 (B). Note: AS03, tocopherol, oil-in-water emulsion-based Adjuvant System; CI, confidence intervals; TIV, inactivated trivalent influenza vaccine; GMT, geometric mean titer; N, number of subjects in the cohort with data available at time-point; Year 1, 2008/09; Year 2, 2009/10; Influenza A strains were A/Brisbane/59/2007 (H1N1 strain) and A/Uruguay/716/2007 (H3N2 strain); Influenza B strains were B/Brisbane/3/2007 (Victoria lineage) in Year 1 and B/Brisbane/60/2008 (Yamagata lineage) in Year 2.

Descriptive immunogenicity in Year 1 and Year 2 is shown in Table 2. At Day 21 in Year 1, GMTs in the AS03-TIV and TIV groups, respectively, were 89.1 and 69.9 for A/H1N1, 285.6 and 172.3 for A/H3N2, and 633.5 and 484.8 for Influenza B (Fig. 2). A similar pattern of response as that observed in Year 1 was observed for GMTs in Year 2 against influenza A strains, whereas GMTs in Year 2 were lower for influenza B at 199.2 and 171.3 in the AS03-TIV and TIV groups, respectively.

**Table 2.** Hemagglutination-inhibition-based immunogenicity in Year 1 and Year 2 in the per-protocol immunogenicity cohort.

		SCR n/N; % (95% CI)		SPR n/N; % (95% CI)		SCF N; value (95% CI)	
		AS03-TIV	TIV	AS03-TIV	TIV	AS03-TIV	TIV
A/H1N1							
Year 1	Day 0	—	—	641/2417; 26.5 (24.8, 28.3)	632/2397; 26.4 (24.6, 28.2)	—	—
	Day 21	1410/2417; 58.3 (56.3, 60.3)	1137/2397; 47.4 (45.4, 49.5)	2081/2422; 85.9 (84.5, 87.3)	1833/2408; 76.1 (74.4, 77.8)	2417; 5.8 (5.5, 6.1)	2397; 4.6 (4.3, 4.8)
Year 2	Day 0	—	—	835/1932; 43.2 (41.0, 45.5)	848/1942; 43.7 (41.4, 45.9)	—	—
	Day 21	776/1931; 40.2 (38.0, 42.4)	662/1941; 34.1 (32.0, 36.3)	1668/1936; 86.2 (84.5, 87.7)	1555/1952; 79.7 (77.8, 81.4)	1931; 3.2 (3.1, 3.4)	1941; 2.9 (2.8, 3.0)
A/H3N2							
Year 1	Day 0	—	—	788/2417; 32.6 (30.7, 34.5)	782/2397; 32.6 (30.7, 34.5)	—	—
	Day 21	2119/2417; 87.7 (86.3, 89.0)	1775/2397; 74.1 (72.2,75.8)	2293/2422; 94.7 (93.7, 95.5)	2146/2408; 89.1 (87.8, 90.3)	2417; 16.4 (15.6, 17.3)	2397; 9.9 (9.4, 10.5)
Year 2	Day 0	—	—	1335/1932; 69.1 (67.0, 71.2)	1224/1942; 63.0 (60.8, 65.2)	—	—
	Day 21	1023/1931; 53.0 (50.7, 55.2)	852/1941; 43.9 (41.7, 46.1)	1897/1937; 97.9 (97.2, 98.5)	1828/1953; 93.6 (92.4, 94.6)	1931; 4.4 (4.2, 4.7)	1941; 3.5 (3.3, 3.7)
B strain							
Year 1	Day 0	—	—	1962/2416; 81.2 (79.6, 82.7)	1936/2397; 80.8 (79.1, 82.3)	—	—
	Day 21	1685/2416; 69.7 (67.9, 71.6)	1428/2397; 59.6 (57.6, 61.5)	2420/2422; 99.9 (99.7, 100)	2397/2408; 99.5 (99.2, 99.8)	2416; 7.4 (7.1, 7.8)	2397; 5.9 (5.6, 6.2)
Year 2	Day 0	—	—	1431/1933; 74.0 (72.0, 76.0)	1431/1942; 73.7 (71.7, 75.6)	—	—
	Day 21	794/1932; 41.1 (38.9, 43.3)	697/1941; 35.9 (33.8, 38.1)	1902/1938; 98.1 (97.4, 98.7)	1875/1953; 96.0 (95.0, 96.8)	1932; 3.4 (3.2, 3.6)	1941; 3.0 (2.9, 3.2)

AS03, tocopherol-based oil-in-water emulsion Adjuvant System; CI, confidence interval; TIV, inactivated trivalent influenza vaccine; N, number of subjects in the cohort; n, number of subjects fulfilled definition of outcome parameter; Year 1, 2008/09; Year 2, 2009/10; Influenza A strains were A/Brisbane/59/2007 (H1N1 strain) and A/Uruguay/716/2007 (H3N2 strain); Influenza B strains were B/Brisbane/3/2007 (Victoria lineage) in Year 1 and B/Brisbane/60/2008 (Yamagata lineage) in Year 2; SCR, seroconversion rate defined as the proportion of subjects with post-vaccination antibody titer of ≥1:40, or pre-vaccination titer of ≥1:10 and ≥4-fold increase post-vaccination; SPR, seroprotection rate defined as the proportion of subjects with antibody titer ≥1:40; SCF, seroconversion factor defined as geometric mean of the ratio between post-vaccination and pre-vaccination reciprocal HI titers.

In Year 1, at Day 21 in the AS03-TIV group, the SCRs and SPRs for the three strains were ≥58.3% and ≥85.9%, respectively, and in the TIV group were ≥47.4% and ≥76.1%, respectively. At Day 21 in Year 2, in the AS03-TIV group, the SCRs and SPRs were ≥40.2% and ≥86.2%, respectively, and in the TIV group were ≥34.1% and ≥79.7%, respectively (Table 2).

### Immunogenicity persistence at Day 180

**Figure 3.**
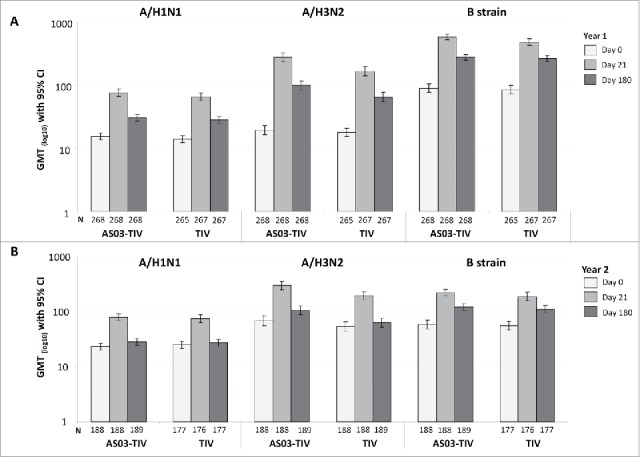
Day 21 and 180 hemagglutination-inhibition-based GMTs in the per-protocol immunogenicity persistence cohorts in Year 1 (A) and Year 2 (B). Note: AS03, tocopherol, oil-in-water emulsion-based Adjuvant System; CI, confidence intervals; TIV, inactivated trivalent influenza vaccine; N, number of subjects in the cohort with data available at time-point; GMT, geometric mean titer; Year 1, 2008/09; Year 2, 2009/10; Influenza A strains were A/Brisbane/59/2007 (H1N1 strain) and A/Uruguay/716/2007 (H3N2 strain); Influenza B strains were B/Brisbane/3/2007 (Victoria lineage) in Year 1 and B/Brisbane/60/2008 (Yamagata lineage) in Year 2.

**Figure 4.**
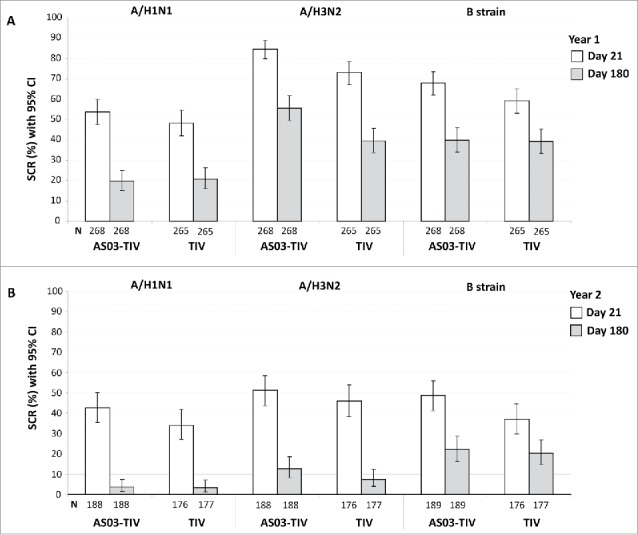
Day 21 and 180 hemagglutination-inhibition-based SCRs in the per-protocol immunogenicity persistence cohorts in Year 1 (A) and Year 2 (B). Note: AS03, tocopherol, oil-in-water emulsion-based Adjuvant System; CI, confidence intervals; TIV, inactivated trivalent influenza vaccine; N, number of subjects in the cohort with data available at time-point; Year 1, 2008/09; Year 2, 2009/10; Influenza A strains were A/Brisbane/59/2007 (H1N1 strain) and A/Uruguay/716/2007 (H3N2 strain); Influenza B strains were B/Brisbane/3/2007 (Victoria lineage) in Year 1 and B/Brisbane/60/2008 (Yamagata lineage) in Year 2; SCR, seroconversion rate defined as the proportion of seronegative subjects at baseline with post-vaccination titer of ≥1:40, or pre-vaccination titer of ≥1:10 and ≥4-fold increase post-vaccination.

In the per-protocol immunogenicity persistence cohort in Year 1 at Day 180 in the AS03-TIV group, the SPRs against A/H1N1, A/H3N2, and influenza B were 55.2%, 78.4%, 99.6%, respectively, and in the TIV group were 50.2%, 69.7%, and 100.0%, respectively. In Year 2 at Day 180 in the AS03-TIV group, the SPRs against A/H1N1, A/H3N2, and influenza B were 47.1%, 86.2%, 93.1%, respectively, in the TIV group were 48.0%, 76.8%, and 90.4%, respectively. In Year 1 in the AS03-TIV group at Days 21 and 180, respectively, GMTs were 75.4 and 30.4 for A/H1N1, 275.0 and 97.7 for A/H3N2, and 573.4 and 274.6 for influenza B; GMTs with in the TIV group at Days 21 and 180, respectively, were 64.5 and 28.1 for A/H1N1, 165.0 and 64.0 for A/H3N2, and 478.5 and 262.3 for influenza B (Fig. 3). The SCRs in the per-protocol immunogenicity persistence cohort from Day 0 to 180 are shown in Figure 4. In Year 1 at Day 180, SCRs for the 3 strains ranged between 19.8% and 55.6% in the AS03-TIV group and between 20.8% and 39.6% in the TIV group, which was higher than SCRs observed at Day 180 in Year 2 between 3.7% and 22.2% and between 3.4% and 0.3%, respectively.

### HI antibody titers as a correlate of protection against A/H3N2

A total of 4828 subjects from the immunogenicity cohort were included in the correlates of protection analysis, of which most subjects were recruited in the Czech Republic (22.7%), Germany (22.1%), and the US (21.7%). During the 2008/09 peak season, influenza epidemic intensity varied between countries, and A/H3N2 was the predominant circulating strain in 12 out of 15 countries based on national surveillance and attack rates in the study (Table 3). A descriptive analysis of the HI antibody titers is shown in Table 4.

**Table 3.** Season strength, circulating influenza viruses, and attack rates by country.

			Attack rate (%) in the total cohort in each country		Dominant strain	
Country	Number subjects in country in the immunogenicity subset (% of total cohort)	A/H3N2 season strength^†^	AS03-TIV	TIV	National	Study
Belgium	134 (2.77%)	high	1.83	1.84	H3N2	H3N2
Canada	130 (2.69%)	moderate	1.47	1.10	H1N1/Influenza B	H3N2/Influenza B
Czech Republic	1096 (22.69%)	high	1.41	1.88	H3N2	H3N2
Estonia	141 (2.92%)	low	1.25	0.90	H3N2/Influenza B	H3N2
France	128 (2.65%)	high	1.62	1.42	H3N2	H3N2
Germany	1068 (22.11%)	moderate	1.03	1.39	H3N2/Influenza B	H3N2
Mexico	130 (2.69%)	low	1.04	0.99	H3N2/H1N1	Influenza B
Netherlands	126 (2.61%)	high	2.68	3.44	H3N2	H3N2
Norway	133 (2.75%)	high	0.88	1.44	H3N2	H3N2
Poland	144 (2.98%)	high	2.62	2.69	H3N2	H3N2
Romania	143 (2.96%)	low	1.47	1.08	H3N2	H3N2
Russia	140 (2.90%)	low	1.06	1.28	H3N2/H1N1	H3N2
Taiwan	139 (2.88%)	low	0.27	0.40	H1N1/Influenza B	H1N1
United Kingdom	130 (2.69%)	high	1.18	1.80	H3N2	H3N2
United States	1048 (21.70%)	moderate	0.51	0.78	H1N1/Influenza B	Influenza B

† Based on national surveillance data and study surveillance based on the review of the Adjudication Steering Committee for the Influenza peak season (2008–2009 influenza season)

**Table 4.** Descriptive statistics HI titers against A/H3N2 in the immunogenicity subset.

Time point	N (n missing)	Mean HI titer (SD)	Min – max HI titer
Day 0			
AS03-TIV	2456	42.63 (94.995)	(5–1810)
TIV	2447	42.48 (89.791)	(5–1280)
Day 21			
AS03-TIV	2456	638.6 (1112.343)	(5–20480)
TIV	2447	449.3 (968.72)	(5–20480)
Day 180			
AS03-TIV	280 (2176)	265.57 (492.528)	(5–5120)
TIV	274 (2173)	174.44 (300.594)	(5–2560)

AS03, tocopherol-based oil-in-water emulsion Adjuvant System; TIV, inactivated trivalent influenza vaccine SD, standard deviation; HI, hemagglutination inhibition

At baseline, 3244/4814 (67.39%) subjects in the immunogenicity subset had a HI titer of ≥1:40 against A/H3N2, and 1570/4814 (32.61%) had a titer of <1:40.

Of the 2422 subjects in the AS03-TIV group, A/H3N2 infection was confirmed in 18 subjects (attack rate: 0.74%), and of the 2408 subjects in the TIV group, A/H3N2 infection was confirmed in 42 subjects (attack rate: 1.74%). A total of 2939 subjects were recruited from regions with low or moderate viral circulation, and 1891 subjects from regions with high viral circulation. Among subjects exposed to low or moderate viral circulation, there were 20 confirmed cases of A/H3N2 infection (attack rate: 0.68%), and among those exposed to high viral circulation, there were 40 confirmed cases of A/H3N2 infection (attack rate: 2.12%).

**Figure 5.**
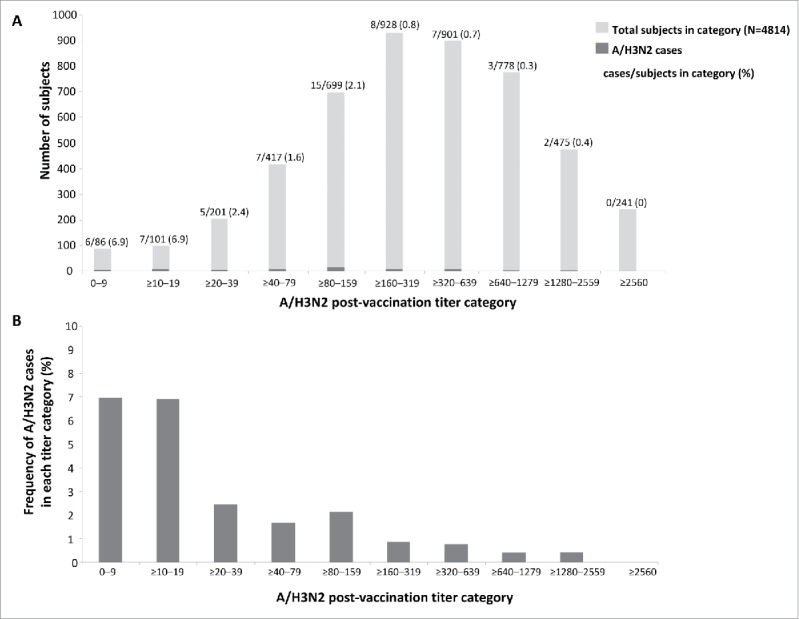
Number of subjects in each titer category and number of A/H3N2 cases (A) and proportion of subjects in each titer category with PCR-confirmed A/H3N2 infection (B) in the immunogenicity subset.

The frequency of A/H3N2 cases and post-vaccination HI antibody titers against A/H3N2 is shown in Figure 5. Among 391/4830 (8.1%) subjects with post-vaccination HI titers of <1:40, 18/391 (4.6%) subjects had PCR-confirmed A/H3N2 infection; among 4439/4830 (91.9%) subjects with post-vaccination titers of ≥1:40, 42/4439 (0.95%) had PCR-confirmed A/H3N2 infection.

**Figure 6.**
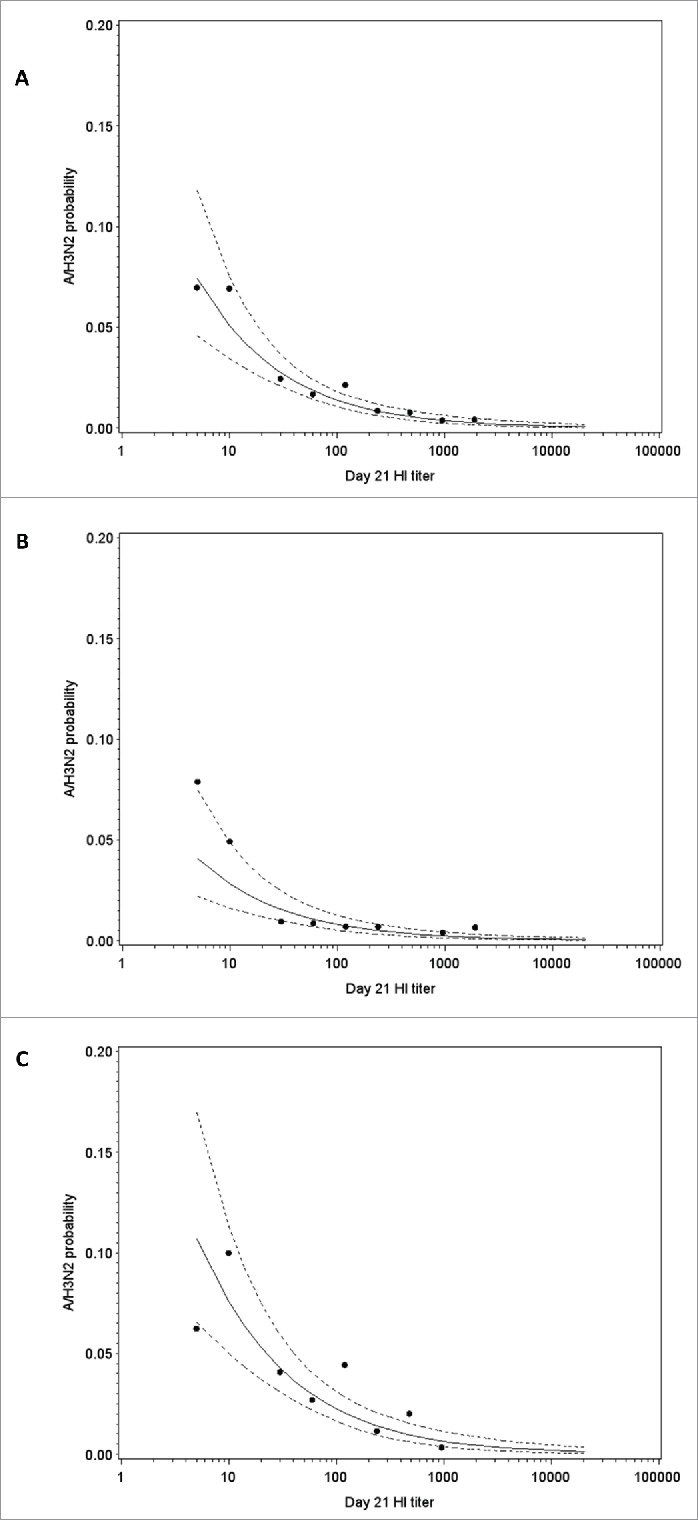
A/H3N2 HI antibody titer and estimated risk of A/H3N2 influenza infection overall (A), in a low/moderate season (B) and in a high season (C) in the immunogenicity subset. Note: Points represent the observed proportions of cases and the dotted curve show 95% confidence interval; HI, hemagglutination-inhibition.

Post-vaccination log titers and season strength were selected for inclusion in the model, suggesting that there is a relationship between post-vaccination HI antibody titers against A/H3N2 and the risk of A/H3N2 infection (Table 5). The model allowed us to estimate that a 4-fold difference in HI titer was associated with a 53% (95% CI: 41%, 63%) decrease in the infection odds. The odds ratio estimate associated to the season strength (high versus low/moderate) was 0.283 (95% CI: 0.164, 0.488). Risk of A/H3N2 infection in a low/moderate and a high season as a function of post-vaccination log-titers are shown in Figure 6. Removing season strength from the model had little impact on the estimated relationship between post-vaccination HI antibody titers against A/H3N2 and the risk of A/H3N2 infection.

**Table 5.** Parameter estimates of the logistic regression model obtained after variable selection.

Parameter	Estimate (SE)	95% Confidence Interval
Intercept	−2.28 (0.44)	[−3.15, −1.42]
Post-vaccination log titers	−1.27 (0.20)	[−1.66, −0.87]
Season Strength	1.04 (0.28)	[0.50, 1.59]

SE, standard error

## Discussion

AS03-TIV and TIV elicited strong HI antibody responses against each vaccine strain in people aged ≥65 years. The manufacturing consistency of 3 lots of AS03-TIV based on HI antibody responses was demonstrated. Twenty-one days after one dose of vaccine in Year 1 and after another dose of vaccine in Year 2, the immunogenicity of both AS03-TIV and TIV fulfilled licensure criteria for influenza vaccines.^11^

Here we describe the immunogenicity findings from the Phase 3 *Influence65* trial, which was based on a subset of 5187 subjects, of which 60% of in each vaccine group were seropositive (HI antibody titer ≥1 :10) before vaccination in Year 1. After vaccination, HI antibody responses were robust, although some strain dependent differences were observed.

After vaccination in Year 1, SCRs against the B strain (Victoria lineage) were 67.9% with AS03-TIV and 59.2% with TIV, and were lower in Year 2 against the B strain (Yamagata lineage) at 48.7% and 36.9%, respectively. For the B strain, the 95% CI for the SPR was ≥60% and for the SCR was ≥30% (fulfilling licensure criteria) in both vaccine groups in both years at Day 21, and SCRs and SPRs remained above this threshold at Day 180 in Year 1. We observed differences in HI antibody responses against the 2 influenza A strains. Immune responses overall were weakest against A/H1N1 in the AS03-TIV and TIV groups at Day 21 in Year 1 (SCRs, 58.3% and 47.4%, respectively). Day 21 SCRs against A/H3N2 were robust in the AS03-TIV and TIV groups in Year 1 (87.7% and 74.1%, respectively), yet were slightly lower in Year 2 (53.0% and 43.9%, respectively). In both years in both vaccine groups, licensure criteria were fulfilled at Day 21 for A/H3N2, and SPRs remained above the licensure threshold at Day 180 in both years in both vaccine groups. SCRs for A/H3N2 at Day 180 remained above the Day 21 licensure threshold in Year 1, but not in Year 2.

A possible explanation for the lower SCR against A/H3N2 in Year 2 than in Year 1 is the effect of baseline antibody titers on immune responses to vaccination. Various studies have shown that pre-vaccination titers, resulting from natural exposure or previous vaccination, can affect the immunogenicity of subsequent seasonal influenza vaccines.^12-15^ In our study, in the immunogenicity cohort, in Year 1, the rate of influenza vaccination in the previous season was 75% and in Year 2 was 100% (i.e. due to study vaccination during Year 1), and during both season in the study A/H3N2 was the predominant circulating strain. It is possible that vaccination in Year 1 or natural exposure to A/H3N2 could have had a negative impact on the immune response in Year 2.

The immunogenicity subset included subjects from all 15 countries in the *Influence65* trial and the wide regional spread of the population is a strength of the trial. However, 66.5% of the immunogenicity subset were recruited in Germany (n = 1068), Czech Republic (n = 1096), and US (n = 1048), which was based the need to limit variation in the population in order to compare immunogenicity between vaccine lots to assess the consistency of the manufacturing process. The results of the *Influence65* trial suggest that AS03-TIV improved protection compared with TIV against influenza A infection (secondary analysis, relative efficacy 17.5%) and influenza A/H3N2 infection in particular (*post-hoc* analysis, relative efficacy 22.0%).^10^ In the immunogenicity subset, the A/H3N2 attack rate was 0.74% in the AS03-TIV group and 1.74% in the TIV group. The estimated relative efficacy between the vaccine groups in the immunogenicity subset appeared high (∼57%), and well above the point estimate (12.16%) in the overall study population. No explanation for this phenomenon has been found.

The relatively high rate of antigenic drift observed with A/H3N2 is thought to be associated with an increased risk of complications and death by this influenza subtype compared with influenza B strains and A/H1N1 in older adults.^16-18^ For example, based on analysis of US hospital discharge records collected for 22 seasons (1979–2001), severe influenza outcomes were much more frequent in the elderly population than among younger adults and children, and the rates of primary pneumonia and influenza-related hospitalization tended to be higher overall in years when A/H3N2 predominated.^16^ In the 2008/09 influenza season in the countries in our study, A/H3N2 was the predominant strain, including high epidemic intensity in Czech Republic and moderate epidemic intensity in Germany. In an analysis of influenza strains in the total vaccinated cohort from the *Influence65* trial, 498 virus samples were subtyped including A/H3N2 (380 viruses), A/H1N1 (29 viruses), B/Yamagata (23 viruses), and B/Victoria (66 viruses), and all but 3 of the A/H3N2 strains were matched to the vaccine strains based on HA1 domain nucleic acid sequence.^19^

Current licensure criteria for influenza vaccines are based on immunogenicity measures that assume post-vaccination HI antibody titers above a defined threshold will be sufficient to prevent influenza in the population.^11^ However, it has been suggested that whereas HI titers may offer a guide to vaccine efficacy, absolute titer may not correlate directly with protection, and serological measures may not be an adequate surrogate for protection.^20-23^ The HI antibody titer threshold of 1:40 is generally recognized as corresponding to a 50% reduction in the risk of influenza, which is based on a challenge study in adults conducted by Hobson et al in 1972.^24^

A previous modeling study based on 15 studies reporting HI titers corresponding to different influenza vaccine strains, supports the reliability of antibody titers as correlates of protection in adults; the study showed that whereas protection increased at titers ≥1:10, a titer of 1:30 was associated with a 50% reduction in the risk of influenza, and a titer of 1:50 was 90% protective.^25^ A clinical study of adults aged 18 to 49 y showed that the cut-off titer for protection against A/H1N1 was as low as 1:30,^26^ although other studies suggest that the thresholds for protection are higher in children and older adults. For example, in children aged 6 to 72 months, a titer of 1:110 was correlated with a 50% protection rate against A/H3N2, and 80% protection was achieved at 1:330.^20^ In a study of adults aged ≥50 years among which post-vaccination HI titers in the population overall were above levels considered to be protective, among subjects with confirmed A/H3N2 infection, 90.9% had extremely low HI titers (≤1:9) against A/H3N2.^27^

However, in elderly populations the currently available vaccine efficacy data in people aged ≥65 years are from the active-controlled *Influence65* trial, and a large randomized trial of high-dose vs. standard dose TIV (Fluzone™; Sanofi Pasteur), which included about 31,989 subjects in the 2011/12 and 2012/13 seasons in the US and Canada. The correlates of protection analysis was based on a randomly selected subset and showed that HI titres of 1:40 corresponded with 50% protection for A/H3N2 cases that were antigenically matched to the vaccine, whereas HI titres of 1:203 to 1:437 were needed for 50% protection in A/H3N2 cases with poor vaccine match.^23^ In the *Influence65* correlates of protection analysis we assessed the distribution of HI titers and the distribution of A/H3N2 cases, and showed that among subjects with post-vaccination HI titers of <1:40, 4.6% had A/H3N2 infection, compared with 0.95% of subjects with post-vaccination titers of ≥1:40. Next we performed a logistic regression, and confirmed a relationship between post-vaccination HI antibody titers against A/H3N2 and the risk of A/H3N2 infection. A 4-fold difference in post-vaccination HI antibody titers against A/H3N2 was associated with a 2-fold decrease in the odds of A/H3N2 infection. However, it should be noted that the population was elderly, and these findings may not extrapolate to younger populations.

It has been previously suggested that HI-based surrogates may be unsuitable for older people because they do not take into account declines in cell-mediated immune responses.^7^ It is recognized that multiple components of immune function are affected by the aging process including impairment of CD4 and CD8 effector and memory T cell responses, and as such, assessments of immune responses to vaccines in older populations should include both humoral and cell-mediated responses, with the clinical goal of increasing both to levels which are correlated with protection.^7,11,14,20,29^

Although our modeling analysis provides an important insight into correlates of protection in elderly people, a limitation of the study was that the *Influence65* trial was not initially planned to include a correlate of protection evaluation. A barrier to evaluating correlates of protection is that most influenza vaccine trials are not powered for this type of assessment. Moreover, in an active control trial, a large proportion of subjects may have high titers and be well protected whereas assessment of a correlate involves contrasting those with low titers and high rates of disease with those with high titers and low rates of disease, and there may be relatively few of the former in an active control trial such as influenza vaccine trials in elderly populations.

In summary, in this large randomized study of people aged ≥65 years, AS03-TIV and TIV elicited strong antibody responses against each vaccine strain 21 d post-vaccination and antibody persistence 6 months post-vaccination were strongest for A/H3N2 and lowest for A/H1N1. A model based on HI titers and A/H3N2 attack rates suggests that a single threshold value for antibody titers within the current definition of seroprotection may not demonstrate adequate protection in older people.

## Methods

### Design and objectives

This Phase 3, randomized, observer-blind study was conducted to assess the efficacy, immunogenicity and safety of an AS03-adjuvanted TIV compared with a non-adjuvanted TIV during the 2008/09 (Year 1) and 2009/10 (Year 2) influenza seasons. The co-primary objective of vaccine efficacy of AS03-TIV versus TIV for the prevention of influenza A and/or B in Year 1, and secondary objectives including vaccine efficacy against clinical outcomes, and reactogenicity and a safety summary, have been reported elsewhere.^32^ Here we describe the co-primary objective of immunogenic lot-to-lot consistency of 3 lots of AS03-TIV, and secondary immunogenicity measures of HI antibody titers at Day 21 and Day 180 post-vaccination in each year.

Here we report immunogenicity in a subset of the Phase 3 population including 5187 subjects from Belgium, Canada, Czech Republic, Estonia, France, Germany, Mexico, Norway, Poland, Romania, Russia, Taiwan, the Netherlands, the United Kingdom, and the United States. The study protocol was approved by Independent Ethics Committees and/or local or central Institutional Review Boards, and was conducted in accordance with Good Clinical Practice, the principles of the Declaration of Helsinki, and all regulatory requirements of participating countries. ClinicalTrials.gov, NCT00753272.

### Subjects

Eligible subjects were men and women aged ≥65 years who were not hospitalized or bedridden, and were without acute illness. Subjects were community-based or lived in a retirement home that allowed mixing in the community. Exclusion criteria included subjects who received any influenza vaccine after February 2008, or vaccination in the previous 3 y with an investigational adjuvanted candidate seasonal or pandemic influenza vaccine. The study protocol was amended in September 2009 to permit vaccination against the human A(H1N1)pdm09 pandemic strain if given at least 14 d before or after study vaccination. All subjects provided informed written consent.

### Vaccines

The vaccines were AS03-TIV (0.7 mL) or TIV (0.5 mL, *Fluarix*™); the Adjuvant System contained squalene and 5.93 mg α-tocopherol in an oil-in-water emulsion (AS03_B_ formulation). Both vaccines were manufactured by GlaxoSmithKline Biological SA (Rixensart, Belgium). Each vaccine dose contained 15 µg of hemagglutinin antigen (HA) for each of the World Health Organization's recommended strains (total 45 µg HA); in both seasons the influenza A strains were A/Brisbane/59/2007 (H1N1 strain) and A/Uruguay/716/2007 (H3N2 strain), and the influenza B strain was B/Brisbane/3/2007 (Victoria lineage) in 2008/09, and B/Brisbane/60/2008 (Yamagata lineage) in 2009/10.

Randomization was implemented with an internet-based system provided by GSK. A blocking scheme was used to randomly assign subjects (1:1) at each site to receive AS03-TIV or TIV. Subjects who were assigned to receive AS03-TIV were further randomly assigned (1:1:1) to one of 3 vaccine lots. Subjects were stratified by age: 65–74 y or 75 y or older. Within both age strata, the randomization algorithm used a minimisation procedure accounting for study center and whether participants lived in a retirement home. Some centers only recruited subjects for the immunogenicity subset, in some centers the first recruited subjects were entered in the immunogenicity subset until the subset was fulfilled, and some centers did not recruit in the immunogenicity subset.

Vaccines were administered intramuscularly in the deltoid muscle region of the non-dominant arm by non-blinded personnel who took no further part in the study procedures; observers and subjects were blind to vaccine allocation. Subjects were scheduled to receive one dose of vaccine in Year 1 and a second dose of the same vaccine in Year 2.

### Objectives

The co-primary objectives of the Phase 3 study were the assessment of relative efficacy of AS03-TIV and TIV and the assessment of the lot-to-lot consistency of 3 consecutive lots of AS03-TIV in a subset of subjects. Lot-to-lot consistency was based on HI assay antibody titers (Geometric Mean Titer [GMT] ratio) against each vaccine strain at Day 21 after the first vaccination in the consistency cohort. Secondary objectives were to assess HI antibody titers at Day 0 and Day 21 for all subjects in the immunogenicity subset (immunogenicity cohort), and at Day 180 in each year in a further subset of subjects (immunogenicity persistence cohort).

### Immunogenicity

Antibody titers against the 3 vaccine strains in each year were measured using a validated micro-titer HI assay as previously described.^28^ HI assay-based antibody responses were described as the anti-log of the arithmetic mean of the log-10 transformed titers (GMTs), seroprotection rate (SPR; proportion of subjects with post-vaccination titer ≥1:40), seroconversion rate (SCR; proportion of seronegative subjects at baseline with post-vaccination titer of ≥1:40, or pre-vaccination titer of ≥1:10 and ≥4-fold increase post-vaccination); and seroconversion factor (SCF; geometric mean of the ratio between pre-vaccination and post-vaccination reciprocal HI titers). Subjects were considered seropositive if they had a pre-vaccination antibody titer of ≥1:10 for a given vaccine strain.

### Statistics

Reactogenicity and safety during the post-vaccination period were to be assessed in a target sample of 6000 subjects (reactogenicity and safety cohort), in order to have around 3000 subjects exposed to AS03-TIV in the safety/reactogenicity analysis. Of the 6000 subjects, 5226 were to be included in the immunogenicity cohort and 600 of these subjects were included in the immunogenicity persistence cohort. The target sample size for the co-primary lot-to-lot consistency analysis was 1749 of the immunogenicity cohort, including 583 subjects per vaccine lot. In order to limit variability in the population for the lot-to-lot consistency assessment, these 1749 subjects were recruited in 3 predefined countries (Czech Republic, Germany and US). Additional subjects for the immunogenicity cohort were recruited over the remaining 12 countries participating in the study, in order to have a representation of each country in case unexpected vaccine efficacy findings would have warranted immunogenicity assessment per country.

Some centers only recruited subjects for the immunogenicity subset, in some centers the first recruited subjects were entered in the immunogenicity subset until the subset was fulfilled, and some centers did not recruit in the immunogenicity subset. Given the additional operational workload of obtaining serum samples and the need to limit variation in the population to compare immunogenicity between vaccine lots to assess the consistency of the manufacturing process, this cohort was recruited from centers in Germany (n = 1068), Czech Republic (n = 1096), and US (n = 1048). The centers were selected based on their high recruitment potential. The immunogenicity persistence subset was allocated from the subjects in the lot-to-lot consistency cohort.

Per-protocol (PP) analyses were performed for each cohort: consistency cohort; immunogenicity cohort (Year 1 and Year 2); and immunogenicity persistence cohort (Year 1 and Year 2), including subjects who met eligibility criteria, complied with the protocol, received any dose of either vaccine, and for whom data were available for a given endpoint. Immunogenicity data (GMTs, SPRs, SCRs, and SCFs) were summarized using descriptive statistics with a 2-sided 95% confidence interval (CI) based on the PP cohorts. GMT ratios were calculated for each AS03-TIV lot comparison (lot 1 vs. lot 2; lot 2 versus lot 3; lot 1 vs. lot 3) using an ANCOVA model. The ANCOVA model included the vaccine group as fixed effect and the pre-vaccination log-transformed titer as regressor. Lot-to-lot consistency was demonstrated if for each vaccine strain the adjusted GMT ratio of the 2-sided 95% CI was within 0.67 and 1.5.

### Correlate of protection analysis

In a *post-hoc* analysis, a statistical regression model was used to assess the relationship between post-vaccination HI antibody titers against A/H3N2 and laboratory-confirmed A/H3N2 influenza attack rates in the Phase 3, vaccine efficacy trial (*Influence65*). Among the 43,695 subjects in the *Influence65* trial, there were 590 PCR-confirmed cases of influenza, including 375 cases caused by A/H3N2, which was the most common influenza virus detected overall.^10^

## Descriptive analysis

All of the covariates used in the analysis were: male or female, age, seasonal influenza vaccination history within previous 2 years, A/H3N2 infection status by the end of the study season, pre- (Day 0) and post-vaccination (Day 21) HI antibody titers against A/H3N2, pre-vaccination A/H3N2 seroprotection status (HI titer ≥1:40), vaccine received (AS03-TIV or TIV), and ‘strong season’ or ‘low/moderate season’. Influenza infection exposure (season strength) was based on national surveillance data and attack rates in the study, as assessed by the Adjudication Steering Committee for the influenza peak season in the *Influence65* trial. Season strength was used as an indicator of the subjects' exposure to the virus. The committee included experts in the field of influenza and influenza vaccination who were independent of the study investigators and the study sponsor. Peak season was defined as the period during the study with the highest incidence of any matching or drift influenza strain relative to the vaccine strains, which was determined *post-hoc* based on national surveillance data and/or study data.

A descriptive analysis of these variables was performed. For continuous variables, the number of observations, mean, standard deviation, and minimum and maximum values were computed. For HI antibody titers, GMTs and their coefficient of variation were also calculated after a log10 transformation. Frequency statistics, including counts and proportions were obtained for the categorical variables. The proportion of subjects with laboratory-confirmed A/H3N2 influenza was calculated for each dilution factor of the post-vaccination HI antibody response against A/H3N2.

## Statistical modeling

The probability of an A/H3N2 disease occurrence was modeled with a logistic regression considering pre-vaccination immunity state (titer ≥1:40 defined as ‘protected’), Day 21 post-vaccination A/H3N2 log titers, gender, history of vaccination (vaccination 1 and 2 y before study start), vaccine received, and season strength as explanatory variables. A manual stepwise variable selection was performed based on the Bayesian information criterion to select the best combination of covariates to describe the disease occurrence. Odds defined as the probability of experiencing an A/H3N2 disease occurrence divided by the probability of not experiencing an A/H3N2 disease occurrence were estimated for several subject profiles from this model. Those profiles were compared by computing odds ratios.

## Trademark

*Fluarix*™ is a trademark of the GSK group of companies.

## Influence65 study group

**Principal Investigators**: Belgium: P-H. Arnould, Y. Balthazar, A-H. Batens, H. Coppens, M. De Meulemeester, P. De Witte, L. Devriendt, G. Leroux-Roels, G. Mathot, O. Maury, P. Muylaert, A. Renson, P. Soetaert, L. Tilley, D. Van Riet, S. Vanden Bemden; Canada: N. Aggarwal, F. Blouin, M. Ferguson, B. Lasko, J. McElhaney, S. McNeil, C. Powell, P. Rheault, D. Shu, E. St-Amour; Czech Republic: J. Beran, V. Chocensky; Estonia: I. Koort, K. Maasalu, A. Poder, L. Randvee, S. Rosenthal, M. Stern, J. Talli; France: R. Arnou, C. Bortolotti, X. Duval, R. Ferrier, C. Fivel, J-F. Foucault, F. Galtier, P. Igigabel, O. Launay, D. Saillard, C. Scellier, Ja. Tondut, P. Uge; Germany: E. Beck, F. Burkhardt, A. Colberg, A. Dahmen, R. Deckelmann, H. Dietrich, R. Dominicus, T. Drescher, T. Eckermann, U. Elefant, M. Esen, G. Fahron, S. Fischer, K. Foerster, H. Folesky, U. Gehling, C. Grigat, A. Himpel-Boenninghoff, P. Hoeschele, S. Höltz, B. Huber, S. Ilg, G. Illies, J-P. Jansen, F. Kaessner, D. Kieninger, C. Klein, U. Kleinecke-Pohl, A. Kluge, W. Kratschmann, K H Krause, P Kremsner, J. Kropp, A. Langenbrink, R. Lehmann, A. Linnhoff, A. Markendorf, G. Meissner, I. Meyer, B. Moeckesch, M. Mueller, S. Mueller, G. Neumann, C. Paschen, G. Plassmann, H-H. Ponitz, A. Preusche, A. Rinke, H. Samer, T. Schaberg, F. Schaper, I. Schenkenberger, J. Schmidt, B. Schmitt, H. Schneider, M. Schumacher, T. Schwarz, H-D. Stahl, K. Steinbach, U. Steinhauser, J. Stockhausen, B. Stolz, N. Toursarkissian, K. Tyler, J. Wachter, H. G. Weber, K. Weyland, D. Wolf, K. Zitzmann; Mexico: C. Aranza Doniz, M.L. Guerrero, E. Lazcano-Ponce, A. Mascareñas de Los Santos, N. Pavia-Ruz, G. M. Ruiz-Palacios; Netherlands: G. A. van Essen, J.H. Richardus, H. Rumke; Norway: S. Elle, A. Holmberg, H. O. Høivik, T. Kjærnli, P. Norheim, A. Tandberg; Poland: W. Gadzinski, J. Holda, E. Jazwinska-Tarnawska, T. Lepich, R. Lysek, H. Nowakowska, M. Orzechowska, Z. Szumlanska; Romania: A. Abaitancei, S. Orban, F. Vasilache, D. Toma; RussianFederation: I. Osipova, O. Perminova, V. Semerikov; Taiwan: S-J. Hwang, P-C. Yang; UK: E. Abdulhakim, I. Pavel-Knox, H. Shaw; United States: M. Blatter, D. Boos, B. Bowling, S. Bowman, D. Brune, S. Christensen, T. Christensen, L. Civitarese, H. EL Sahly, J. Earl, J. Ervin, B. Essink, A. R. Falsey, G. Feldman, T. Fiel, C. Fogarty, S. Folkerth, S. E. Frey, D. Fried, G. Gibson, M. Hall, W. Harper, S. Hull, J. Jacobson, J. Jacqmein, J. Lawless, C. Lucasti, T. Poling, G. Raad, G. Ramsbottom, K. Reisinger, E. Riffer, J. Rosen, E. Ross, J. Rubino, S. Sperber, H. Studdard, J. Thrasher, M. Turner, M. Van Cleeff, L. Wadsworth, J. Yakish

**GSK Vaccines Clinical Study Support**: A. Caplanusi, C. Claeys, J.-M. Devaster, B. Innis, M. Kovac, L. Oostvogels, C. Van Der Zee

**Laboratory partners**: F. Allard, S. Durviaux, N. Houard, T. Ollinger, K. Walravens

**Statistical analysis partners**: W. Dewé, C. Durand, M. El Idrissi, M. Oujaa

**Members of the Independent Data Monitoring Committee**: J. Claassen, A. Grau, R. Konior (chair), F. Verheugt, N Stouffer

**Members of the Adjudication Committee**: M. Betancourt-Cravioto, D. Fleming, K. Nichol, W. J. Paget (chair)

## Abbreviations

AS03: tocopherol, oil-in-water emulsion-based Adjuvant System
AS03-TIV: AS03-adjuvanted inactivated trivalent influenza vaccine
CI: confidence interval
GMT: geometric mean titers
HA: hemagglutinin antigen
HI: hemagglutination-inhibition
SE: standard error
SCF: seroconversion factor
SCR: seroconversion rate
SPR: seroprotection rate
TIV: inactivated trivalent influenza vaccine
